# Genomic and functional approaches point to potential determinants of neonatal *K. pneumoniae* sepsis

**DOI:** 10.1128/spectrum.04118-25

**Published:** 2026-04-27

**Authors:** Manisha Rout, Kusum Rani, Jharana Mahanta, Subasini Majhi, Soumya Sibani Sahoo, Satyaranjan Mallick, Dharitri Mohapatra, Tapas Kumar Som, Ashoka Mahapatra, Soumitra Mohanty, V. Samuel Raj, Saroj Kant Mohapatra

**Affiliations:** 1 Biotechnology Research and Innovation Council - National Institute of Biomedical Genomics (BRIC-NIBMG) 214255, Kalyani, West Bengal, India; 2 Regional Centre for Biotechnology, NCR Biotech Science Cluster 682813 , Faridabad, Haryana, India; 3 Department of Biotechnology, SRM University, Delhi-NCR, Sonepat, Haryana, India; 4 Department of Microbiology, Srirama Chandra Bhanja Medical College and Hospital, Cuttack, Odisha, India; 5 Department of Paediatrics, Srirama Chandra Bhanja Medical College and Hospital, Cuttack, Odisha, India; 6 Department of Neonatology, All India Institute of Medical Sciences 410775, Bhubaneswar, Odisha, India; 7 Department of Microbiology, All India Institute of Medical Sciences 410775, Bhubaneswar, Odisha, India; Icahn School of Medicine at Mount Sinai, New York, New York, USA

**Keywords:** neonatal sepsis, *Klebsiella pneumoniae*, genomics, multidrug resistance, virulence, siderophores, yersiniabactin

## Abstract

Neonatal sepsis remains a major cause of global mortality, yet the contribution of pathogen-level traits to clinical outcomes is poorly understood. We combined whole-genome sequencing with phenotypic and functional assays to evaluate whether genomic features of *Klebsiella pneumoniae*, particularly the stealth siderophore yersiniabactin (*ybt*), are associated with clinical outcomes in neonates. Thirty-three bloodstream isolates were profiled for antimicrobial resistance (AMR), virulence genes, and sequence type. Phenotypic assays assessed antimicrobial susceptibility and biofilm formation, and host-pathogen interactions were examined using a human airway epithelial cell (A549) model. *Ybt*-positive isolates showed increased adhesion and intracellular proliferation in A549 cells compared to ybt-negative isolates and were enriched among isolates recovered from non-surviving neonates. Genomic clustering revealed frequent co-occurrence of *ybt* with multidrug resistance determinants in high-risk sequence types, such as ST14 and ST231. Traditional phenotypes, such as AST and biofilm formation, did not segregate by outcome, whereas *ybt* presence showed a stronger association with outcome in this cohort. These findings suggest that siderophore-mediated iron acquisition may enhance epithelial fitness and contribute to adverse outcomes in neonatal sepsis. Our study highlights the value of integrating genomic and functional bacterial features into neonatal sepsis research and illustrates the potential of pathogen-based markers to complement host-focused prognostic strategies.

**IMPORTANCE:**

Neonatal sepsis caused by *Klebsiella pneumoniae* remains a major clinical challenge, yet the pathogen traits that contribute to fatal outcomes are poorly understood. In this study, we analyzed bloodstream isolates from 33 neonates, including 13 who did not survive, and identified genomic features enriched in strains from non-survivors. The siderophore yersiniabactin (*ybt*), which enables iron acquisition and helps bacteria withstand host immune defenses, was consistently present in these isolates. By integrating genomic profiling with laboratory phenotypes and epithelial cell infection assays, we show that *ybt*-positive strains exhibit enhanced epithelial fitness and are associated with adverse clinical outcomes in this cohort. These findings highlight the importance of incorporating pathogen genomic and functional markers into neonatal sepsis research, surveillance, and future risk-stratification strategies.

## INTRODUCTION

Sepsis remains a major global health challenge, responsible for an estimated 48.9 million cases and 11 million deaths annually (1). This burden disproportionately affects low- and middle-income countries (LMICs), particularly India, where diagnostic limitations and healthcare disparities exacerbate underreporting. Neonates are especially vulnerable; the risk of sepsis is highest during the first 28 days of life. Global estimates attribute nearly 5 million neonatal sepsis cases and 800,000 deaths per year (2). South Asia accounts for a significant share, with over 60% of neonatal sepsis cases occurring within 72 h of birth and mortality approaching 33% in culture-confirmed cases (3). In India alone, neonatal sepsis constitutes 14.3% of all Neonatal Intensive Care Unit (NICU) admissions (4).

*Klebsiella pneumoniae* is a predominant pathogen in neonatal sepsis globally, and especially in India, often exhibiting extensive drug resistance (5). Isolates frequently harbor extended-spectrum β-lactamases (ESBLs) and carbapenemase genes, including *blaNDM* and *blaOXA-48*-like variants, limiting treatment options. Surveillance programs, such as BARNARDS and NeoOBS, underscore that *K. pneumoniae* is not only highly prevalent but also often resistant to first-line and last-resort antibiotics (6).

Beyond resistance, *K. pneumoniae* has evolved potent virulence mechanisms, including capsular polysaccharides (K-locus), LPS O-antigens, siderophores, and adhesins. Certain lineages exhibit hypervirulence, classically associated with community-acquired invasive infections, but increasingly seen in hospital settings. Of these, stealth siderophores—notably *yersiniabactin (ybt*) and *aerobactin (iuc*)—enable immune evasion by circumventing host iron-sequestration defenses such as lipocalin-2 (7).

Historically, resistance and virulence were considered mutually exclusive traits found in separate *K. pneumoniae* lineages (8). However, recent studies reveal an alarming convergence: resistant clones, such as ST14, ST23, and ST258, have acquired virulence loci, particularly *ybt*, through horizontal gene transfer (9). Integrative conjugative elements (ICEkp) are central to this co-acquisition, resulting in strains with both antimicrobial resistance and enhanced pathogenic potential.

Predicting outcome in neonatal sepsis remains a clinical priority, yet current risk assessment relies mainly on host factors and antimicrobial susceptibility reports. However, emerging genomic evidence suggests that pathogen-level determinants, particularly iron-acquisition systems such as ybt, may influence invasiveness and clinical outcome (5, 10, 11). Although mechanistic studies demonstrate ybt-mediated iron acquisition, lipocalin-2 evasion, and copper detoxification, there is still no direct experimental evidence linking ybt-positive neonatal isolates to poor clinical outcome (12–14). Thus, the role of ybt in outcome-associated virulence remains biologically plausible but clinically under-validated. Moreover, traditional phenotypic assays, such as AST or biofilm tests, alone fail to capture the nuanced contribution of virulence genes to disease progression and do not reflect complex host-pathogen interactions. Coupled with emerging genomic evidence, this highlights the need for an integrated pathogen-centric framework that unites genomic profiling, phenotypic traits, and functional assays to elucidate and potentially predict neonatal sepsis outcomes.

To address these gaps, we investigated 33 neonatal bloodstream isolates of *K. pneumoniae* through a combined genomic, phenotypic, and functional framework. Whole-genome sequencing was used to profile antimicrobial resistance (AMR) and virulence loci, including siderophore gene clusters. Phenotypic assays evaluated antimicrobial susceptibility and biofilm formation, and host-pathogen interaction assays in human airway epithelial (A549) cells were used to assess bacterial adhesion and intracellular persistence. Guided by these complementary data sets, we further asked whether specific genomic/functional signatures—particularly those involving siderophores such as yersiniabactin—may help explain differences in clinical outcome, including mortality, among neonates with sepsis.

## MATERIALS AND METHODS

### Bacterial isolates

Thirty-three clinical isolates of *Klebsiella pneumoniae* were obtained from blood cultures between 2018 and 2023 (Table 1). Nineteen isolates were collected from S.C.B. Medical College & Hospital, Cuttack, Odisha, with ethics approval from the Institutional Ethics Committee (IEC Appln. No. 1170, dated 29-10-2022), and six isolates from AII India Institute of Medical Sciences, Bhubaneswar, Odisha, with ethics approval from the Institutional Ethics Committee (Ref. number: T/EMF/Micro/22/32, dated 12-09-2022). The remaining eight isolates were kindly provided by the laboratory of Prof. V. Samuel Raj, SRM University, Delhi-NCR, Sonepat. To ensure robust assessment of pathogen-specific factors influencing neonatal bloodstream infection outcomes, we adopted a multi-center sampling strategy, collecting isolates from three geographically distinct neonatal units in India. Geographic and institutional diversity was an essential design consideration because antimicrobial resistance patterns, circulating *K. pneumoniae* lineages, and NICU-level infection control practices are known to vary substantially across regions. Multi-center sampling, which is well established in large neonatal sepsis studies from India (15, 16), therefore enhances the representativeness of our isolate collection, minimizes center-specific bias, and improves the validity and generalizability of pathogen-outcome associations identified in this study. All isolates were obtained from blood cultures of neonates with clinically suspected and laboratory-confirmed sepsis based on standardized clinical criteria (age 0–28 days and the presence of at least one of the following signs: temperature instability, respiratory alteration including oxygen support or ventilation, cardiovascular instability, neurological symptoms, gastrointestinal symptoms, or clinician suspicion of sepsis warranting antibiotic therapy). Both term and preterm neonates were included, whereas infants with major congenital anomalies were excluded. The isolates were stored in glycerol stocks at −80°C until further phenotypic and genotypic characterization.

**TABLE 1:** Clinical and microbiological characteristics of neonates infected with *K. pneumoniae* isolates used in this study^*a*^

LabID	Center of collection	Age	Synopsis	Outcome	Resistance status	Biofilm strength	Sequence type	nSG
M001	SCBMCH	4D	Early onset of sepsis	Patient not available for follow-up	XDR	Weak	ST258	1
M002	SCBMCH	2D	Sepsis, low birth weight	Patient not available for follow-up	XDR	Weak	ST258	1
M003	SCBMCH	9D	Sepsis	Patient not available for follow-up	XDR	Weak	ST258	1
M004	SCBMCH	10D	Sepsis	Patient not available for follow-up	MDR	Moderate	ST244	1
M005	SCBMCH	1D	Early onset of sepsis	Patient not available for follow-up	XDR	Moderate	ST231	2
M007	SCBMCH	8D	Sepsis, term/AGA/HIE- II	Patient not available for follow-up	XDR	Moderate	ST2735	1
M008	SCBMCH	8D	Sepsis, term/SGA, shock	Patient not available for follow-up	MDR	Strong	ST2735	1
M010	SCBMCH	3D	Sepsis	Patient not available for follow-up	XDR	Moderate	ST228	1
M011	SRM	<28D	Sepsis	Non-survivor	XDR	Moderate	ST231-1LV	3
M012	SRM	<28D	Sepsis	Non-survivor	XDR	Moderate	ST231	3
M014	SRM	3D	Sepsis	Non-survivor	XDR	Moderate	ST14	2
M015	SRM	1D	Sepsis	Non-survivor	XDR	Moderate	ST231	3
M017	SRM	2D	Sepsis	Non-survivor	XDR	Strong	ST14	2
M018	SRM	3D	Sepsis	Non-survivor	XDR	Strong	ST14	2
M019	SRM	3D	Sepsis	Non-survivor	XDR	Strong	ST14	2
M020	SRM	7D	Sepsis	Non-survivor	XDR	Strong	ST14	2
1NKBL	AIIMS-B	4D	Refractory septic shock/ Early onset sepsis/ELBW/Very preterm/DIC (pulmonary hemorrhage)	Non-survivor	MDR	Weak	ST1991-2LV	2
2NKL	AIIMS-B	3D	Sepsis	Patient not available for follow-up	MDR	Weak	ST1991-2LV	2
4NKBL	AIIMS-B	4D	Twin I/Moderate preterm/Male/Appropriate for dates/EMCS/LBW/Apnea of prematurity/Spontaneous intestinal perforation/Peritonitis/Refractory septic shock/AKI/DIC/Pulmonary hemorrhage	Non-survivor	MDR	Weak	ST23	4
4NKL	AIIMS-B	6D	Sepsis	Non-survivor	MDR	Strong	ST515	2
5NKBL	AIIMS-B	3D	Refractory septic shock/Gastrointestinal perforation/EPT/ELBW/EOS/Bilateral grade III IVH/AKI/GI hemorrhage/DIC	Non-survivor	XDR	Weak	ST91	3
8NKL	AIIMS-B	8D	Sepsis	Non-survivor	PDR	Moderate	ST231	3
M024	SCBMCH	2D	Sepsis	Patient not available for follow-up	MDR	None	ST133	1
M026	SCBMCH	14D	Sepsis	Patient not available for follow-up	Not MDR	Moderate	ST2313-2LV	2
M028	SCBMCH	9D	Sepsis	Patient not available for follow-up	MDR	Moderate	ST2313-2LV	2
M033	SCBMCH	7D	Sepsis	Patient not available for follow-up	MDR	Weak	ST2313-2LV	2
M039	SCBMCH	4D	Sepsis	Patient not available for follow-up	MDR	Weak	ST661-1LV	2
M040	SCBMCH	12D	Sepsis	Patient not available for follow-up	MDR	Weak	ST17	1
M041	SCBMCH	14D	Sepsis	Patient not available for follow-up	MDR	Weak	ST25	3
M042	SCBMCH	5D	Sepsis	Patient not available for follow-up	MDR	Weak	ST1573-3LV	1
M047	SCBMCH	2D	Term/AGA/EOS/HIE-III	Patient not available for follow-up	MDR	Weak	ST23	4
M048	SCBMCH	4D	Sepsis	Patient not available for follow-up	MDR	Weak	ST91	3
M051	SCBMCH	13D	Sepsis	Patient not available for follow-up	MDR	Weak	ST420	4

^*a*^ This table summarizes the clinical metadata and pathogen features for the 33* K. pneumoniae* isolates included in the study. Variables include patient age at onset (in days), center of collection, sepsis synopsis, clinical outcome, and isolate-specific characteristics, such as antimicrobial resistance (MDR/XDR/PDR), biofilm formation strength (qualitatively classified from crystal violet assay), sequence type (ST), and the number of siderophore-associated virulence genes identified from whole-genome sequencing. Clinical outcomes are stratified as “non-survivor” or “patient not available for follow-up.” Resistance status was determined using standard AST profiling. Sequence types were derived using MLST, and siderophore genes were annotated using Kleborate.

### Antimicrobial susceptibility testing (AST)

Susceptibility profiles were determined using the Kirby-Bauer disk diffusion method with Enterobacteriaceae Dodeca disks (HiMedia, India), following the manufacturer instructions. A single colony from nutrient agar (NA) was transferred to Mueller-Hinton agar (MHA) and incubated aerobically at 37°C for 12–14 h. After reaching 0.5 McFarland turbidity (~1.5 × 10⁸ CFU/mL), cultures were swabbed onto fresh MHA plates, and Dodeca disk panels 1 and 2 were applied. Plates were incubated aerobically at 37°C for 16–18 h, and zone diameters were measured using HiMedia antimicrobial zone scales. Results were interpreted according to CLSI guidelines (17, 18), and isolates were classified as multidrug-resistant (MDR) or extensively drug-resistant (XDR) or pandrug-resistant (PDR) per Magiorakos et al. (19).

### Biofilm formation assay

Quantitative biofilm production was assessed using a modified crystal violet microtiter assay (20). Overnight cultures grown in Brain Heart infusion (BHI) broth were standardized to 0.5 McFarland turbidity, and 200 µL was added in eight replicates per strain into 96-well flat-bottom plates. Plates were incubated statically and aerobically at 37°C for 16 h. After washing with sterile Milli-Q water, the wells were stained with 0.1% crystal violet for 15 min. The bound stain was solubilized in 30% glacial acetic acid, and the absorbance was measured at 575 nm. Negative control consisted of sterile BHI while the positive control was *K. pneumoniae* ATCC 700603. Optical density cut-off (ODc) was defined as the mean OD of negative controls + 3 × SD. Isolates were classified as non-, weak, moderate, or strong biofilm producers according to Stepanović et al. (21)

### Genome sequencing and *de novo* assembly

Genomic DNA was extracted using standard Qiagen kits, and purity and concentration were determined by Nanodrop and Qubit. Illumina libraries were prepared using the KAPA Hyper Prep Kit and sequenced on a NovaSeq 6000, while Nanopore libraries were prepared with ONT barcoding kits and sequenced on PromethION and GridION platforms. Standard trimming and quality filtering were applied. Assemblies were generated using Unicycler (22) (Illumina) or Flye with Medaka polishing (Nanopore) (23, 24) and evaluated with QUAST (25). Genotypic profiling, including MLST, K/O loci, AMR, and virulence genes, was performed using Kleborate (26) with hypervirulence defined by established marker genes (27). A detailed methodology for sample processing, quality control, sequencing, and analysis is provided in the File S1 at http://dx.doi.org/10.6084/m9.figshare.31530211.

### Sample clustering and correlation

Sample clustering based on virulence genes and antimicrobial resistance genes was performed using hierarchical clustering on binary presence/absence matrices derived from Kleborate outputs. Clustering was performed with Euclidean distance and complete linkage and visualized using the Heatmap() function from the ComplexHeatmap R package. A correlation matrix was constructed to explore associations between phenotypic AST results and genotypic AMR markers using the corrplot() function from the corrplot R package.

### Cell infection assay

A549 airway epithelial cells (ATCC CCL-185) were maintained in Dulbecco’s Modified Eagle Medium supplemented with 10% fetal bovine serum (FBS) and standard additives. For infection assays, cells were seeded at 2 × 10⁵ cells per well in 24-well plates. Prior to the infection, the old media were replaced with serum- and antibiotic-free media. The optimal multiplicity of infection (MOI = 50:1) was determined using viability assays with *Klebsiella pneumoniae* subsp. *pneumoniae* strain NCTC 9633/ATCC 13883 (Kp13883) across MOIs of 10:1, 50:1, and 100:1. Four clinical isolates and one control strain (Table 2) were used in adhesion and intracellular proliferation assays. These isolates were chosen based on their differential biofilm-forming abilities and the diversity in siderophore gene profiles, including variations in the number and types of siderophore genes present. After a 30-minute infection, non-adherent bacteria were removed by a 1× phosphate buffered saline (PBS) wash. Further, cells were lysed with 0.1% Triton X-100, and colony-forming units (CFUs) from adherent bacteria were enumerated on nutrient agar plates. However, for intracellular proliferation, cells were treated with sub-minimum inhibitory concentration antibiotics (1/4 MIC) and lysed at 0.5–4 h intervals post-infection. CFUs were quantified to assess intracellular proliferation kinetics.

**TABLE 2:** Selected *K. pneumoniae* isolates used for host-pathogen interaction assays^*a*^

LabID	Biofilm strength	Number of siderophore genes (nSG)	Siderophore gene	Antimicrobial concentration for infection assay
M008	Strong	1	*ent*	Amikacin (0.5 µg/mL)
M010	Moderate	1	*ent*	Amikacin (128 µg/mL)
M015	Moderate	3	*ent*,*ybt*,*iuc*	Colistin (0.5 µg/mL)
M017	Strong	2	*ent*,*ybt*	Amikacin (16 µg/mL)
ATCC 13883	Weak	2	*ent*,*ybt*	Amikacin (0.5 µg/mL)

^*a*^ Four clinical isolates were chosen based on their biofilm-forming ability and the number of siderophore-associated virulence genes detected by whole-genome sequencing. *Klebsiella pneumoniae *subsp.* pneumoniae* strain NCTC 9633 (ATCC 13883) was included as a control. The table summarizes each isolate’s biofilm activity (qualitative strength from A₅₇₅), the total number of siderophore genes, and the antimicrobial concentration used during intracellular infection assays in A549 airway epithelial cells. Strains represent both high- and low-virulence profiles, with antibiotic concentrations tailored to reflect minimum inhibitory concentrations (MICs) for effective extracellular clearance during amikacin, or colistin protection assays.

### Reactive oxygen species (ROS) assay

To quantify total intracellular ROS generated, A549 cells were cultured in a 96-well plate and infected with six clinical isolates (M008, M010, M015, M017, 1NKBL, 4NKBL) of *K. pneumoniae* in MOI of 50:1 under serum- and antibiotic-free conditions for defined time intervals (15, 30, 60, and 120 min). The experimental design incorporates controls, including uninfected cells and buffer blanks, to distinguish true intracellular ROS signals from background fluorescence. After each exposure time, the culture supernatant was removed, and cells were incubated with 100 µL DCFH-DA in HBSS to yield the final concentration of 10 µM in each well. The plate was incubated at 37°C with 5% CO_2_ for 15 min in the dark. Fluorescence was measured using a Tecan plate reader at Ex 485 nm / Em 535 nm.

### Statistical analysis

Statistical tests included Shapiro-Wilk and Kruskal-Wallis for biofilm strength comparisons, *t*-tests for adhesion assays, linear regression for modeling adhesion and intracellular proliferation, and for analyzing ROS generation over time and the presence of yersiniabactin. Pearson’s Chi-square tests were applied to evaluate statistically significant association between resistance phenotypes and corresponding genes, as well as between the presence of genes or mutations with outcome. A Least Absolute Shrinkage and Selection Operator (LASSO) logistic regression model was built to predict mortality among neonates based on AST, biofilm formation, AMR and virulence genes, and STs, with receiver operating characteristic curve/area under the curve (ROC/AUC) evaluation performed using the pROC (R package). All analyses were performed in R (v4.1.0) (28).

## RESULTS

### Antimicrobial susceptibility pattern

**Fig 1:**
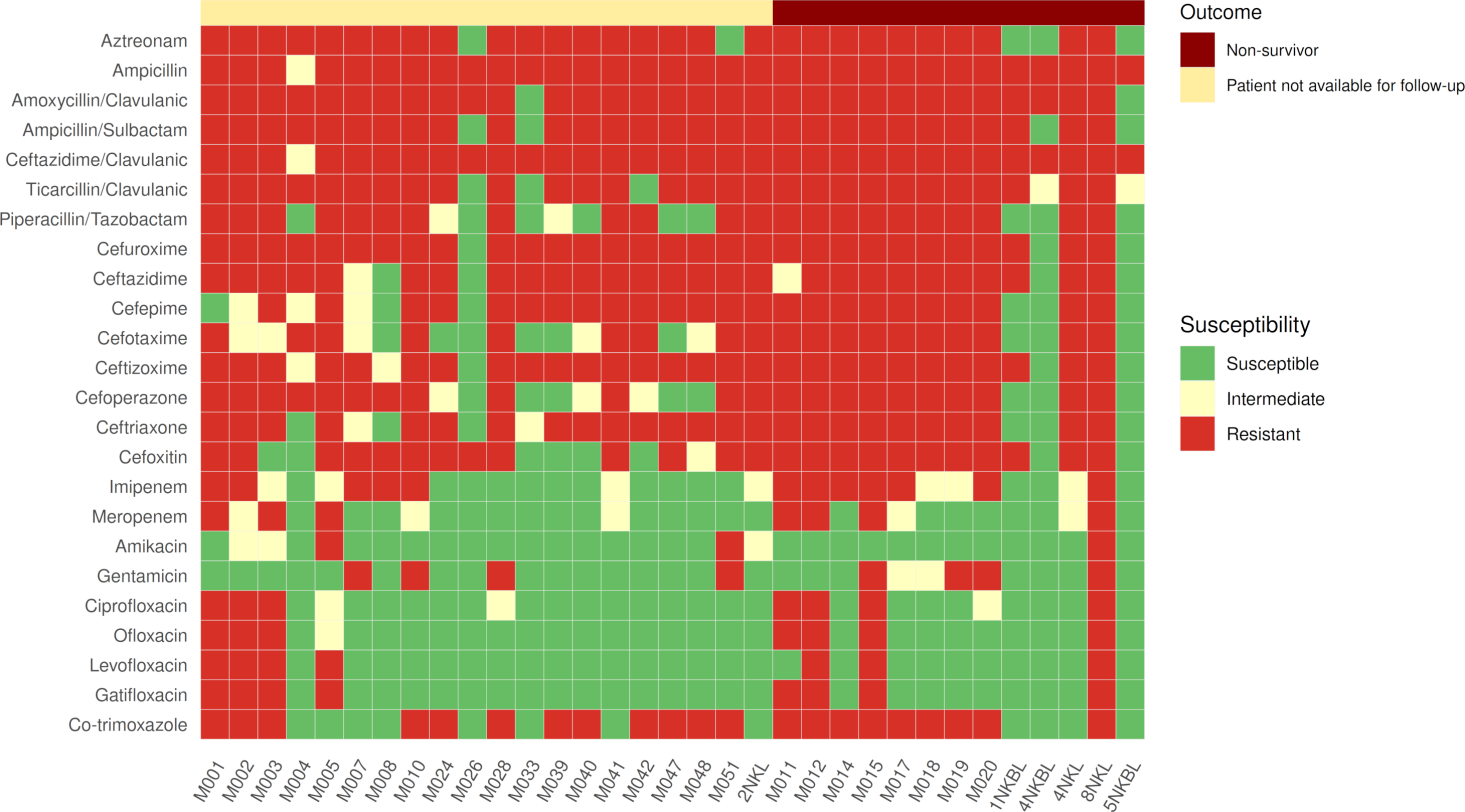
Heatmap showing antimicrobial susceptibility patterns of 33 *K. pneumoniae* isolates tested against a panel of 24 antimicrobials across different classes. Rows represent antimicrobial agents, and columns represent individual isolates. The color scale indicates phenotypic susceptibility: green = susceptible, yellow = intermediate, and red = resistant. The top annotation panel classifies isolates by clinical outcome: dark red = non-survivor, light yellow = patient not available for follow-up. The figure highlights extensive resistance to β-lactams and cephalosporins, with relative susceptibility to aminoglycosides and fluoroquinolones. Overall, the isolates from neonates with sepsis display resistance to multiple antimicrobials.

To assess resistance profiles of the clinical isolates, all 33 *K. pneumoniae* strains were subjected to antimicrobial susceptibility testing. All isolates exhibited multidrug resistance (MDR), with several also displaying extensive drug resistance (XDR) (Table 1). Resistance was most pronounced against β-lactams, β-lactamase inhibitors, and monobactams, followed by second-, third-, and fourth-generation cephalosporins and metabolic inhibitors. In contrast, resistance to aminoglycosides and fluoroquinolones was comparatively lower (<50%) (see Table S4 at http://dx.doi.org/10.6084/m9.figshare.31530211). Notably, β-lactam and extended-spectrum β-lactam resistance were more frequently observed among isolates recovered from non-survivors. Protein synthesis inhibitors (e.g., aminoglycosides) and DNA synthesis inhibitors (e.g., fluoroquinolones) showed the highest *in vitro* susceptibility (Fig. 1).

### Biofilm formation assay

**Fig 2:**
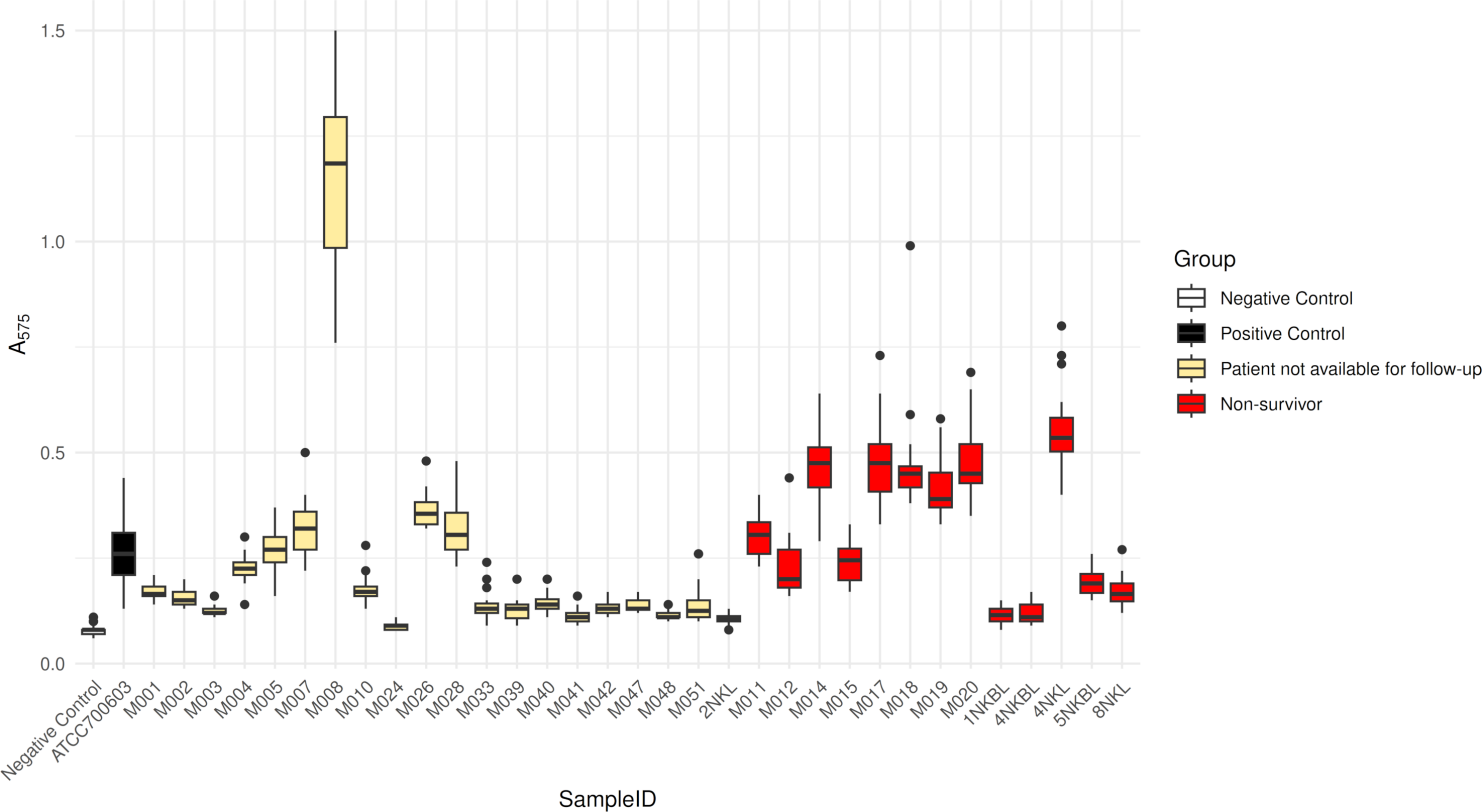
Quantification of biofilm formation in *K. pneumoniae* clinical isolates. Boxplots showing the absorbance values (A₅₇₅) from crystal violet staining as a measure of biofilm biomass formed by 33 *K. pneumoniae* isolates, alongside negative and positive controls. Each box is colored based on clinical outcome: non-survivors (dark red), patients not available for follow-up (light yellow), and controls (white = negative [sterile BHI], black = positive [ATCC 700603]). Higher absorbance values indicate stronger biofilm formation. Notably, isolates from non-survivors exhibited significantly greater biofilm-forming ability compared to other groups, with M008 as an exception among the follow-up unavailable group. Data represent replicate measurements (*N* = 3 biological replicates, *n* = 7 technical replicates) for each sample, and whiskers show interquartile range.

Biofilm formation is a known mechanism that enhances bacterial ability to evade the host immune system as well as the reach of antibiotics. To evaluate this phenotype, we performed a quantitative microtiter biofilm assay. Isolates from non-survivors (*N* = 13) exhibited significantly stronger biofilm formation than those from patients not available to follow-up (*N* = 20), as measured by A_575_ absorbance values (*P* = 0.0000312) (Fig. 2; Table 1). This suggests enhanced persistence capabilities among strains associated with poor outcomes.

### Genomic characterization

**Fig 3:**
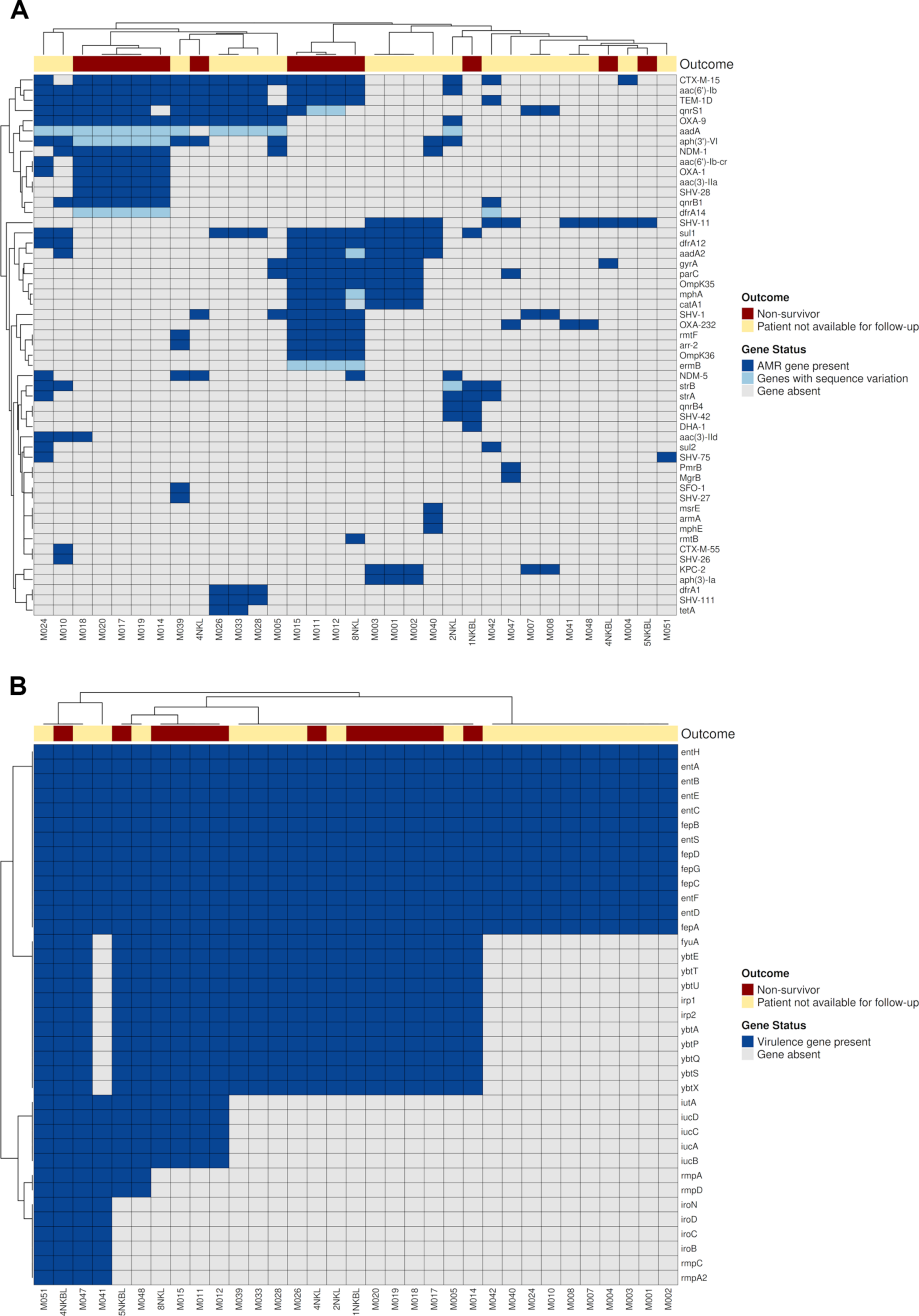
(**A**) Hierarchical clustering of AMR gene profiles in 33 *Klebsiella pneumoniae* isolates. A heatmap depicts the presence and sequence status of antimicrobial resistance (AMR) genes across isolates. Each row represents an AMR gene, and each column represents a clinical isolate. Columns are annotated by clinical outcome: non-survivors (dark red) and “lost to follow-up” cases (light yellow). Gene status is color-coded as follows: present (dark blue), present with sequence variation (light blue), or absent (gray). Clustering was performed using hierarchical clustering (Euclidean distance, complete linkage). The heatmap illustrates the distribution of major AMR determinants, including ESBLs (e.g., *CTX-M-15*, *SHV*) and carbapenemases (*NDM*, *OXA* variants). (**B**) Hierarchical clustering of virulence gene profiles across 33 *Klebsiella pneumoniae* isolates. A heatmap shows the presence or absence of virulence-associated genes. Each row represents a virulence gene, and each column represents a clinical isolate. Columns are annotated by clinical outcome: non-survivors (dark red) and “lost to follow-up” cases (light yellow). Gene status is color-coded as present (dark blue) or absent (gray). Clustering was performed using hierarchical clustering (Euclidean distance, complete linkage). Several isolates from non-survivors carry key siderophore systems, including yersiniabactin (*ybt*), aerobactin (*iuc*), and salmochelin (*iro*).

To characterize the genetic background of the isolates, Kleborate was used to analyze MLST types, K and O loci, and virulence and resistance genes (Tables 1 and 3; Fig. 3A and B). Among non-survivors, ST14 (5/13) and ST231 (4/13) were the predominant sequence types (ST). These isolates also carried multiple hypervirulence determinants, with virulence scores of 1 or 4 (indicating the presence of yersiniabactin (*ybt*) with or without aerobactin (*iuc*)), with additional occurrence of salmochelin (*iro*), and *rmpA/rmpA2*. Most non-survivor isolates, especially those belonging to high-risk clones, such as ST14, ST231, ST23, ST515, and ST91, harbored either an intact or partially truncated hypervirulence cassette, frequently including *iuc1* or *iuc2A*, and an ICEKp-associated yersiniabactin. There were marked differences in antimicrobial resistance genotypes between isolates from fatal cases and those from patients lost to follow-up. Isolates from non-survivors generally carried higher Kleborate resistance scores, indicating the presence of genes encoding extended-spectrum β-lactamases (*CTX-M-15*), carbapenemases (*NDM-1/NDM-5* or *OXA-232*), and 16S rRNA methyltransferases (*rmtF* and *rmtB*), together forming XDR genotypes. In contrast, isolates from outcome-unknown (lost-to-follow-up) neonates displayed heterogeneity. Two resistance genes—*aac(3)-IIa* and *SHV-28*—were carried by one group of isolates from non-survivors, particularly within the ST14 high-risk cluster. Another group of isolates from non-surviving neonates harbored porin-loss mutations (*OmpK35* truncations and *OmpK36* TD insertions), which may synergistically enhance carbapenem resistance.

**TABLE 3:** Genotypic characterization of *Klebsiella pneumoniae* isolates using Kleborate^*a*^

LabID	Virulence score	Yersiniabactin	Aerobactin	Salmochelin	RmpADC	rmpA2	Resistance score	Number of resistance classes	Number of resistance genes	wzi	K locus	K type	O_locus	O type
M001	0	Absent	Absent	Absent	Absent	Absent	2	7	8	wzi154	KL107	Unknown (KL107)	OL2α.2	O2β
M002	0	Absent	Absent	Absent	Absent	Absent	2	7	8	wzi154	KL107	Unknown (KL107)	OL2α.2	O2β
M003	0	Absent	Absent	Absent	Absent	Absent	2	7	8	wzi154	KL107	Unknown (KL107)	OL2α.2	O2β
M004	0	Absent	Absent	Absent	Absent	Absent	1	1	1	wzi50	KL15	K15	OL4	O4
M005	1	ybt 13; ICEKp2	Absent	Absent	Absent	Absent	2	5	6	wzi104	KL51	K51	OL2α.2	O1αβ,2β
M007	0	Absent	Absent	Absent	Absent	Absent	2	2	2	wzi10	KL10	Capsule null	OL2α.1	O2α
M008	0	Absent	Absent	Absent	Absent	Absent	2	2	2	wzi10	KL10	Capsule null	OL2α.1	O2α
M010	0	Absent	Absent	Absent	Absent	Absent	2	7	14	Absent	KL57	K57	OL2α.2	O1αβ,2β
M011	4	ybt 14; ICEKp5 (truncated)	iuc 5 (truncated)	Absent	Absent	Absent	2	10	16	wzi104	KL51	K51	OL2α.2	O1αβ,2β
M012	4	ybt 14; ICEKp5 (truncated)	iuc 5	Absent	Absent	Absent	2	10	16	wzi104	KL51	K51	OL2α.2	O1αβ,2β
M014	1	ybt 9; ICEKp3 (truncated)	Absent	Absent	Absent	Absent	2	6	13	wzi2	KL2	K2	OL2α.1	O1αβ,2α
M015	4	ybt 14; ICEKp5 (truncated)	iuc unknown	Absent	Absent	Absent	2	10	16	wzi104	KL51	K51	OL2α.2	O1αβ,2β
M017	1	ybt 9; ICEKp3 (truncated)	Absent	Absent	Absent	Absent	2	6	14	Absent	KL2	K2	OL2α.1	O1αβ,2α
M018	1	ybt 9; ICEKp3 (truncated)	Absent	Absent	Absent	Absent	2	6	14	wzi2	KL2	K2	OL2α.1	O1αβ,2α
M019	1	ybt 9; ICEKp3 (truncated)	Absent	Absent	Absent	Absent	2	6	18	wzi2	KL2	K2	OL2α.1	O1αβ,2α
M020	1	ybt 9; ICEKp3 (truncated)	Absent	Absent	Absent	Absent	2	6	14	wzi2	KL2	K2	OL2α.1	O1αβ,2α
1NKBL	1	ybt 7; ICEKp7	Absent	Absent	Absent	Absent	0	4	8	wzi364	KL132	Unknown (KL132)	OL2α.1	O1αβ,2α
2NKL	1	ybt 7; ICEKp7 (truncated)	Absent	Absent	Absent	Absent	2	5	9	wzi364	KL132	Unknown (KL132)	OL2α.1	O1αβ,2α
4NKBL	4	ybt 8; ICEKp3 (truncated)	iuc 1	iro 1	rmp 1; KpVP-1 (truncated)	rmpA2_6* −47%	0	1	0	wzi1	KL1	Capsule null	OL2α.2	O1αβ,2β
4NKL	1	ybt 14; ICEKp5	Absent	Absent	Absent	Absent	2	5	7	Absent	KL115	Unknown (KL115)	OL13	O13
5NKBL	4	ybt 10; ICEKp4?	iuc 2A	Absent	rmp unknown	Absent	0	0	0	Absent	KL4	K4	OL2α.1	O2α
8NKL	4	ybt 14; ICEKp5 (truncated)	iuc unknown	Absent	Absent	Absent	2	10	16	Absent	KL51	Capsule null	OL2α.2	O1αβ,2β
M024	0	–	Absent	Absent	Absent	Absent	2	7	17	Absent	KL116	Unknown (KL116)	OL2α.1	O1αβ,2α
M026	1	ybt 13; ICEKp2 (truncated)	Absent	Absent	Absent	Absent	1	7	9	Absent	KL180	Unknown (KL180)	OL2α.3	O1αβ,2γ
M028	1	ybt 13; ICEKp2	Absent	Absent	Absent	Absent	1	6	8	Absent	KL180	Unknown (KL180)	OL2α.3	O1αβ,2γ
M033	1	ybt 13; ICEKp2	Absent	Absent	Absent	Absent	1	7	9	Absent	KL180	Unknown (KL180)	OL2α.3	O1αβ,2γ
M039	1	ybt 15; ICEKp11 (truncated)	Absent	Absent	Absent	Absent	2	6	13	wzi84	KL28	Capsule null	OL2α.1	O2α
M040	0	Absent	Absent	Absent	Absent	Absent	2	5	8	wzi141	KL25	K25	OL5	O5
M041	3	Absent	iuc 1	iro 1	rmp 1; KpVP-1 (truncated)	rmpA2_8* −47%	2	1	1	wzi72	KL2	K2	OL2α.2	O1αβ,2β
M042	0	Absent	Absent	Absent	Absent	Absent	1	6	7	Absent	KL53	Capsule null	OL3γ	O3γ
M047	4	ybt unknown	iuc 1	iro 1	rmp 1; KpVP-1 (truncated)	rmpA2_6* −55%	3	3	1	Absent	KL1	Capsule null	OL2α.2	O1αβ,2α
M048	4	ybt 10; ICEKp4? (truncated)	iuc 2A	Absent	rmp unknown	Absent	2	1	1	Absent	KL4	K4	OL2α.1	O2α
M051	4	ybt 9; ICEKp3	iuc 1	iro 1	rmp 1; KpVP-1 (truncated)	rmpA2_5 −54%	0	0	0	wzi95	KL20	K20	OL2α.1	O1αβ,2α

^*a*^ Kleborate-derived genomic features for 33* K. pneumoniae* isolates, including virulence score and resistance score. Virulence scores indicate the presence of key loci, such as yersiniabactin (*ybt*), aerobactin (*iuc*), colibactin (*clb*, if present). Resistance scores reflect the detection of major antimicrobial resistance determinants, including extended-spectrum β-lactamases (ESBLs) and carbapenemases. These metrics summarize the convergence of AMR and virulence traits and informed the selection of isolates for functional assays.

Kaptive analysis identified diverse K-locus types. Non-survivors were mostly associated with high-risk hypervirulence capsule types commonly associated with hypervirulent lineages (e.g., KL2). In comparison, isolates from the “lost to follow-up” group showed more diverse distribution of K-loci with a predominance of classical MDR-associated types (e.g., KL107, KL180, and KL10). Comparison of O-locus distribution between non-survivor and “lost to follow-up” groups revealed that both groups were dominated by O2 family antigens (OL2α.1 and OL2α.2), which together account for nearly 80% (26/33) of total isolates. Although a small number of rare O-types (OL4, OL13, OL5, OL3γ, OL2α.3) appeared predominantly (6/7) among isolates from “lost to follow-up” neonates, their low frequency provides inadequate evidence of O-antigen type as a driver of disease severity in this cohort.

### Presence of AMR and virulence genes by outcome

To investigate how resistance and virulence gene content aligned with clinical outcomes, we performed unsupervised hierarchical clustering based on the presence/absence matrices from Kleborate (Fig. 3A and B). AMR gene clustering showed that isolates from non-survivor frequently harbored genes conferring resistance to aminoglycosides (*aac(6')-Ib*, *aac(6')-Ib-cr*, *aph(3')-VI*, *aac(3)-IIa)*, fluoroquinolones (*qnrB1*, *qnrS1*), and β-lactams (*OXA-1*, *OXA-9*, *NDM-1*, *TEM-1D*, *CTX-M-15*, *SHV-28*). While specific AMR genes were disproportionately represented among non-survivors, clustering of isolates by AMR genes (presence/absence/sequence variation) failed to segregate the outcome groups. This highlights that AMR gene distribution lacks discriminatory power and does not independently explain clinical divergence. Non-survivors were clustered into four clades based on virulence gene content. However, these clades were small and should be interpreted cautiously. Among non-survivors, one subgroup harbored both *ybt* and *iuc* loci, while another carried *ybt* alone. Only a minority of isolates carried the *rmpA/rmpA2* or *iro* loci. While a few isolates from lost-to-follow-up neonates also carried these genes coding for siderophores and hypermucoviscosity, all isolates carried the *ent* gene.

### Correlation of AMR genotype with phenotype

**Fig 4:**
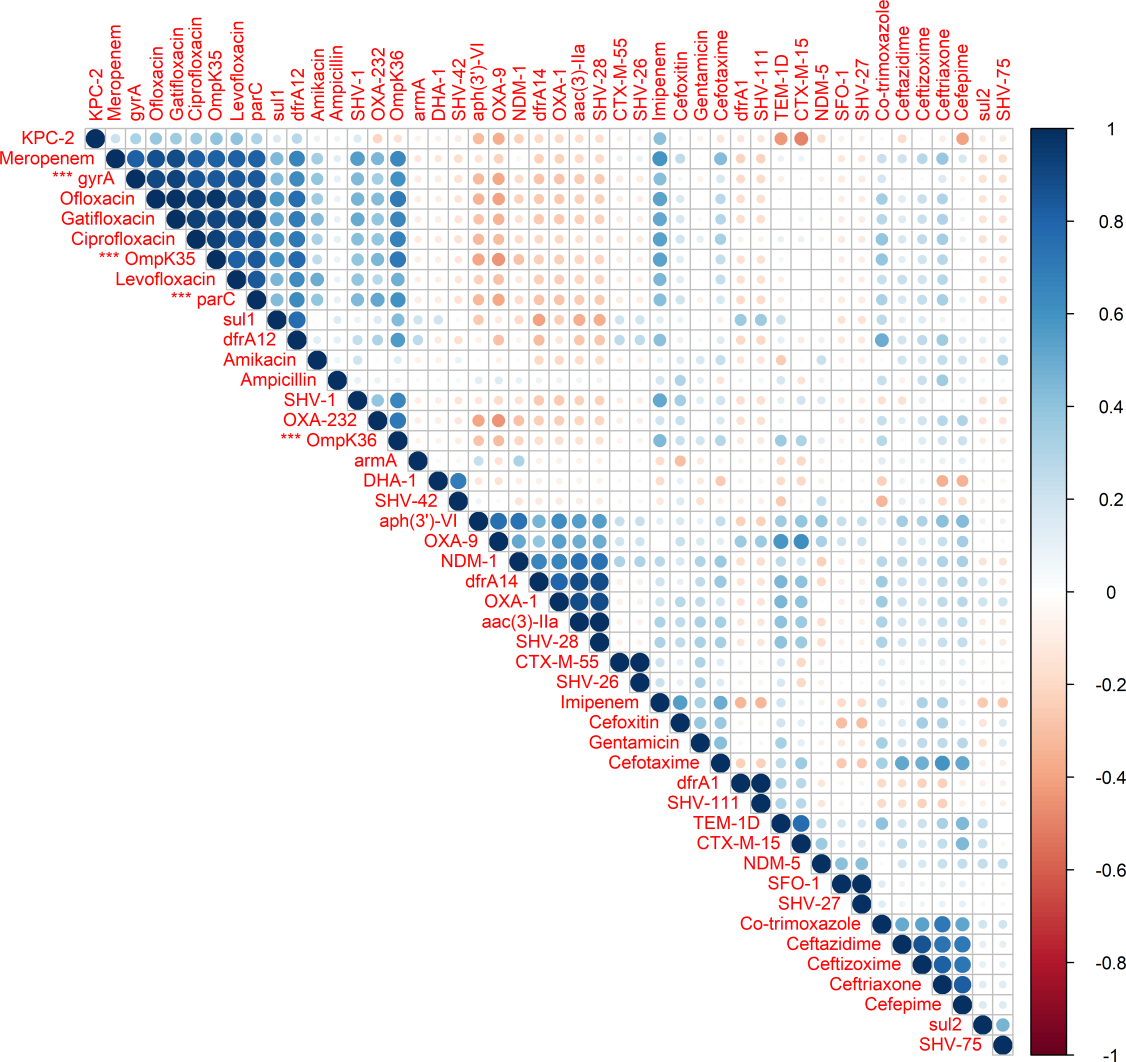
Correlation matrix of phenotypic and genotypic antimicrobial resistance. Pairwise Pearson correlation coefficients between antimicrobial susceptibility phenotypes (AST results) and resistance-associated genes detected by whole-genome sequencing are shown. Each circle represents a correlation value: blue denotes positive correlations and red denotes negative correlations, with color intensity and circle size reflecting the magnitude of correlation (|r|). Antibiotics and AMR genes (red labels) are clustered based on similarity in correlation patterns. Notable associations include correlations between *gyrA*, *parC*, and *OmpK35* with fluoroquinolone and meropenem resistance, and clusters of co-occurring AMR genes, such as *aph(3′)-VI*, *OXA-9*, *OXA-1*, *NDM-1*, *dfrA14*, *aac(3)-IIa*, and *SHV-28*.

To assess the agreement between genotypic and phenotypic resistance, we constructed a correlation matrix (Fig. 4). Strong positive correlations (*P* < 0.05) were observed between fluoroquinolone resistance and the gyrA/parC mutations, as well as between carbapenems and *OmpK35/OmpK36* mutations. Additionally, co-occurrence of multiple AMR genes (*aph(3')-VI*, *OXA-9*, *OXA-1*, *NDM-1*, *dfrA14*, *aac(3)-IIa*, *SHV-28*) encoding resistance to different antimicrobial classes was observed. This supports the biological plausibility of the observed phenotypic resistance patterns.

### Adhesion, intracellular proliferation, and total ROS generation in A549 cells during infection

**Fig 5:**
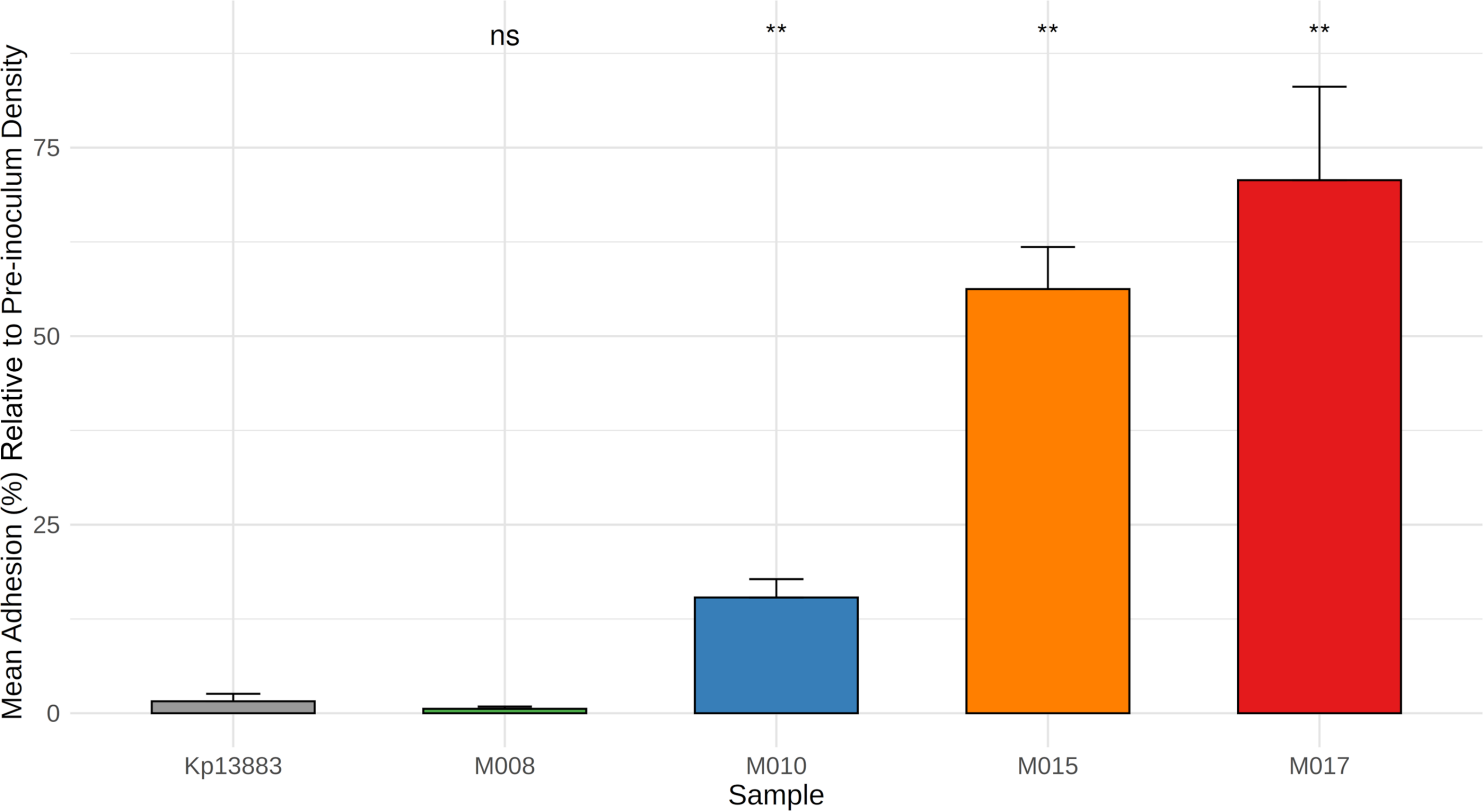
Adhesion of *K. pneumoniae* clinical isolates to human airway epithelial (A549) cells. Bar plot showing the mean percentage of adherent bacteria relative to the initial inoculum density (measured by CFU) for five *K. pneumoniae* isolates (*N* = 5 strains, *n* = 6 technical replicates). Kp13883 (gray) is a laboratory control strain; M008 (green) and M010 (blue) are *ybt*-negative isolates from outcome-unknown (lost-to-follow-up) neonates; M015 (orange) and M017 (red) are *ybt*-positive isolates from non-surviving neonates. A549 cells were infected at an MOI of 50:1 for 30 min, washed to remove non-adherent bacteria, and lysed for CFU quantification. Bars represent mean ± SEM. Asterisks denote statistical significance compared to the Kp13883: ns = not significant; ** = *P* < 0.005 (Welch *t*-test). *Ybt*-positive isolates (M015, M017) showed higher adhesion to A549 cells relative to *ybt*-negative strains.

Bacterial attachment to epithelial surfaces is an important first step in establishing infection. Adhesion assays in A549 cells showed significantly higher attachment (*P* < 0.005) for all test isolates, except M008, compared with the reference strain Kp13883 (Fig. 5). Notably, the *ybt*-positive isolates M015 and M017 exhibited the strongest adhesion, whereas M008—despite being a strong biofilm producer—displayed poor attachment, demonstrating that biofilm formation and epithelial adhesion are distinct phenotypic traits. Linear regression analysis showed a strong positive association between the number of siderophore loci carried by an isolate and its adhesion capacity (*P* < 0.001), consistent with enhanced epithelial interaction among siderophore-rich strains.

**Fig 6:**
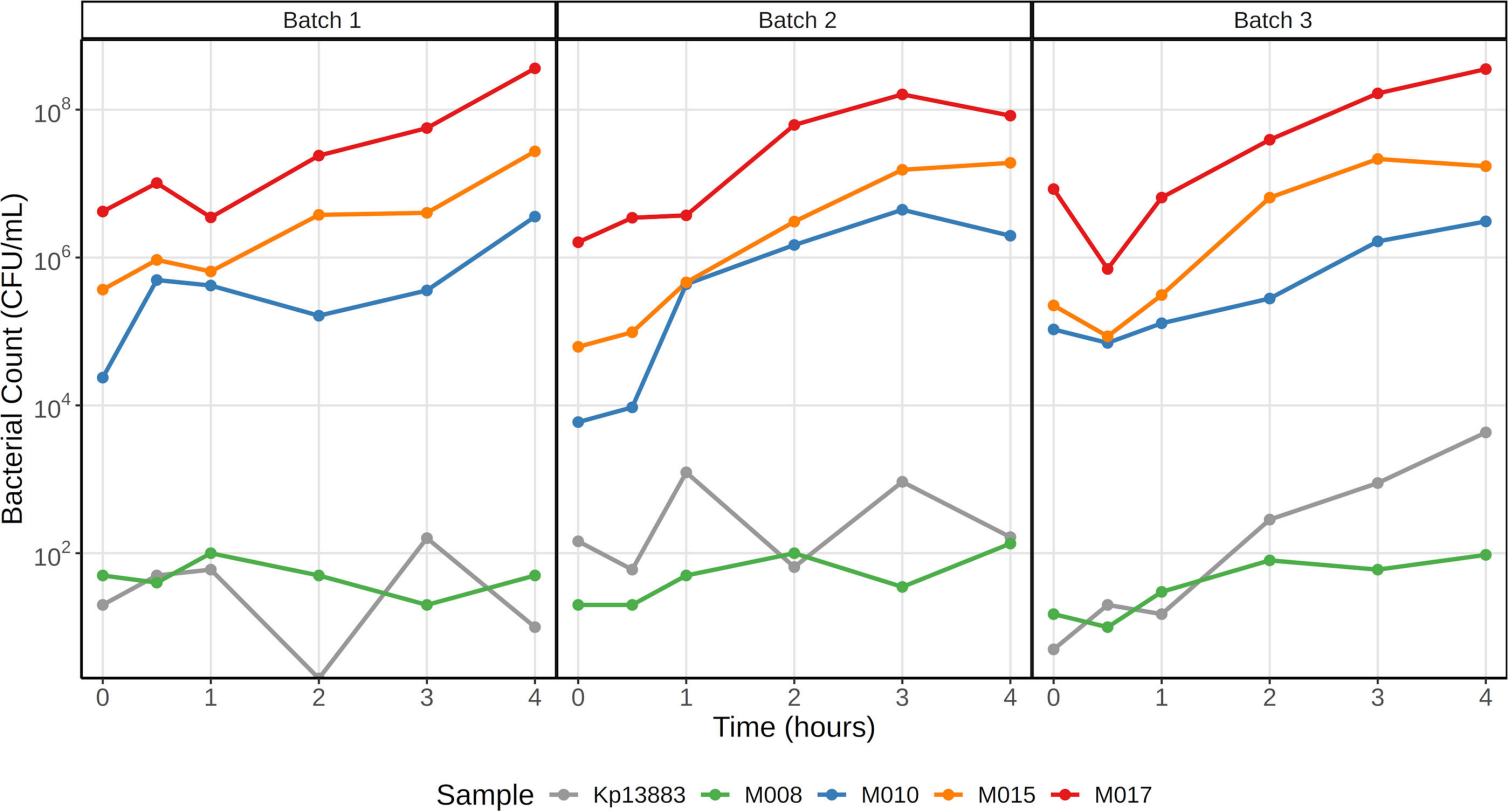
Intracellular proliferation dynamics of *Klebsiella pneumoniae* isolates in human airway epithelial (A549) cells. Line plot showing changes in intracellular bacterial load (CFU/mL, log₁₀ scale) over 4 h for five *K. pneumoniae isolates*: Kp13883 (gray, laboratory control), M008 (green) and M010 (blue) from outcome-unknown (lost-to-follow-up) neonates, and M015 (orange) and M017 (red) from non-survivors. A549 cells were infected at an MOI of 50:1 for 1 h, followed by antibiotic treatment (amikacin; colistin used for amikacin-resistant strains) for 30 min to remove extracellular bacteria. Intracellular CFUs were quantified at defined time points from 0.5 to 4 h after antibiotic treatment. Data represent three biological replicates per strain. *Ybt*-positive isolates (M015, M017) showed higher intracellular proliferation, whereas *ybt*-negative isolates (M008, M010) and the control strain Kp13883 demonstrated limited intracellular survival.

To assess intracellular survival and proliferation, intracellular CFUs were quantified over a 4-hour period following antibiotic protection (Fig. 6). M015 and M017 (both *ybt*-positive) showed the strongest and most sustained intracellular growth, while M010 exhibited only modest increases over time. In contrast, M008 and the reference strain Kp13883 showed minimal or no proliferation. Linear regression analysis revealed a highly significant association between the number of siderophore loci carried by an isolate and its intracellular growth (*P* = 3.51 × 10⁻¹⁰). The differing behavior of the two *ybt*-negative isolates, M008 and M010, highlights that factors other than biofilm capacity contribute to intracellular survival dynamics.

To assess the contribution of reactive oxygen species (ROS) during epithelial infection, total intracellular ROS levels were measured in A549 cells at 15, 30, 60, and 120 min post-infection (see Fig. S1 at http://dx.doi.org/10.6084/m9.figshare.31530211). Across all isolates, intracellular ROS levels showed a significant overall decline during the time course (*P* = 8.73 × 10⁻⁶). At the earliest time point (15 min), *ybt*-positive isolates exhibited significantly lower ROS levels compared with ybt-negative isolates (*P* = 0.00556), indicating a reduced oxidative response early during infection. The subsequent rate of ROS decline also differed by siderophore status, with *ybt*-positive isolates showing a significantly slower decrease in fluorescence intensity (*P* = 0.0098). In contrast, *ybt*-negative isolates M008 and M010 displayed high initial ROS levels followed by rapid reduction—a pattern consistent with their reduced adhesion and intracellular survival phenotypes. Among *ybt*-positive isolates, ROS levels remained relatively stable over time, with one isolate showing a late secondary rise. These patterns suggest that *ybt*-positive isolates are associated with lower early ROS activation and a more stable ROS trajectory during infection, although this does not establish a causal mechanism for intracellular persistence.

### Predictive modeling of clinical outcome

**Fig 7:**
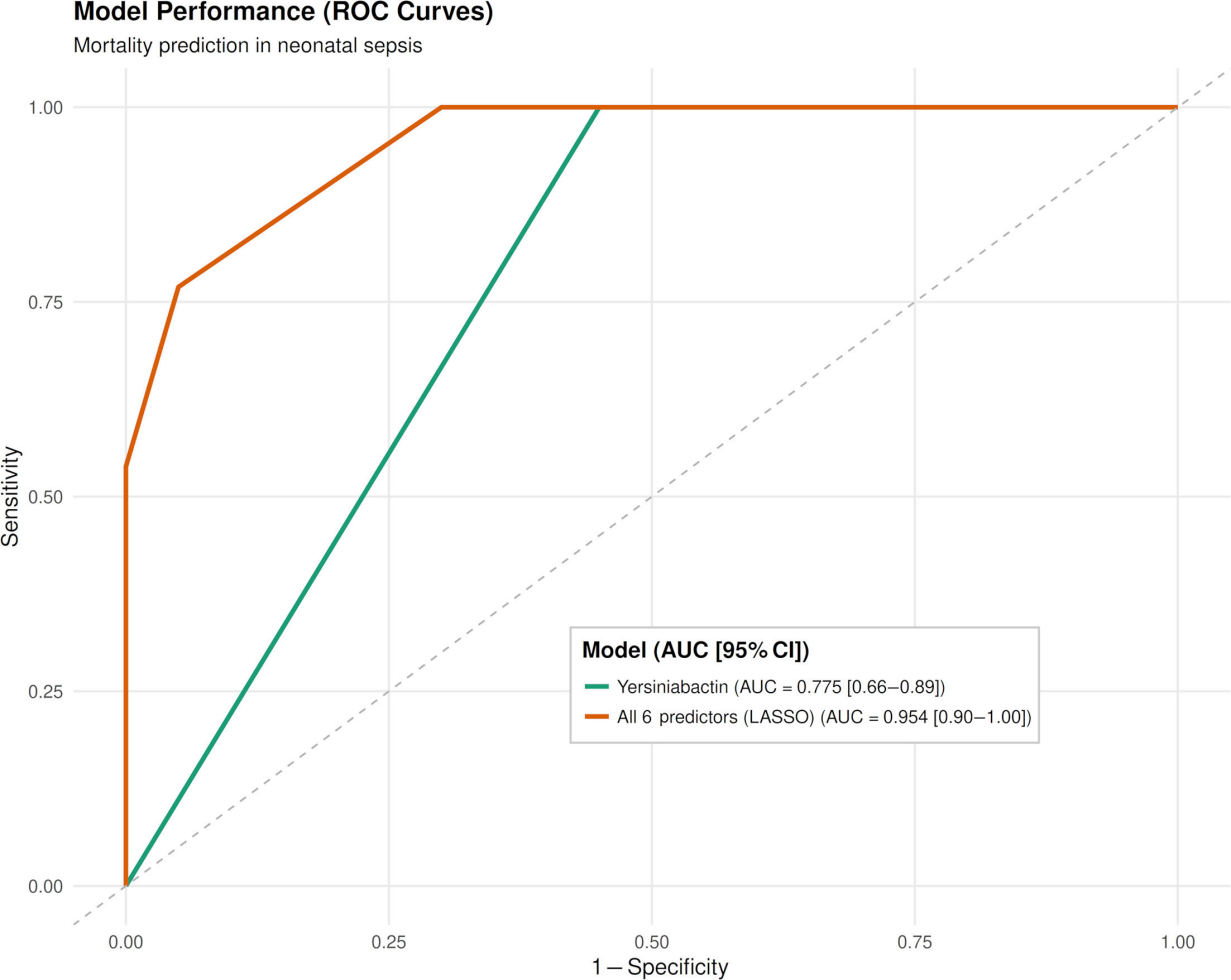
ROC curve of a LASSO-regularized logistic regression model predicting neonatal mortality in *Klebsiella pneumoniae* sepsis. Receiver operating characteristic (ROC) curve shows the performance of a penalized logistic regression model (L1/LASSO) trained on 33 neonatal bloodstream isolates. Predictor variables included antimicrobial susceptibility phenotypes (fluoroquinolone, aminoglycoside, β-lactam/β-lactamase inhibitor), biofilm formation (A₅₇₅), virulence determinants (e.g., yersiniabactin), and sequence type indicators. LASSO was used for variable selection and shrinkage to reduce overfitting. The presence of yersiniabactin alone demonstrated moderate predictive ability with AUC of 0.775 (95% CI: 0.66–0.89). A six-feature model (*ybt* plus five ST categories) achieved excellent discrimination with an AUC of 0.954 (95% CI: 0.90–1.00). These results illustrate the potential of integrating genomic and phenotypic features for risk stratification in neonatal sepsis.

Comparative receiver operating characteristic (ROC) analysis showed progressive improvement in discrimination as additional features were incorporated into the predictive model (Fig. 7). The presence of the yersiniabactin locus alone demonstrated moderate predictive ability (AUC = 0.775). In contrast, the six-feature LASSO-selected model—including *ybt* and five sequence type indicators (ST14, ST231, ST515, ST231-1LV, ST2313-2LV)—achieved excellent discriminatory performance (AUC = 0.954). Yersiniabactin was the strongest predictor retained by LASSO, while specific sequence types (notably ST14) further improved classification accuracy. While these findings illustrate the potential of integrating genomic features for risk stratification in neonatal sepsis, the model should be interpreted as exploratory given the limited sample size.

## DISCUSSION

This study provides critical insights into the stealth siderophore yersiniabactin (*ybt*), a pathogen-associated feature that may be linked with neonatal outcomes in *Klebsiella pneumoniae* sepsis, and provides mechanistic support linking *ybt* to enhanced intracellular survival, immune evasion, and other features relevant to clinical severity. By integrating genomic profiling with functional assays, we demonstrate *ybt*-positive isolates from non-surviving neonates exhibit a distinct fitness advantage in airway epithelial cells, suggesting contributions not fully explained by antimicrobial resistance alone. To extend these mechanistic observations, we additionally integrate genomic and functional features into an exploratory predictive model aimed at identifying pathogen-level traits associated with outcome.

Prior work has shown that siderophore-host interaction shapes the trajectory of *K. pneumoniae* infection. In murine respiratory infection models using isogenic siderophore mutants of *K. pneumoniae*, Bachman et al. (2011 and 2012) (12, 13) demonstrated that *ybt* promotes bacterial growth in the lungs despite host lipocalin-2 activity, while dissemination to the spleen was restricted by transferrin. Additionally, siderophore production during infection has been linked to induction of cellular stress, proinflammatory cytokines, and stabilization of HIF-1α, ultimately leading to bacterial dissemination beyond the primary site of infection (29). Together, these studies highlight the dual role of siderophores in nutrient acquisition and in creating a host niche that is permissive for systemic spread.

Our findings extend these observations to neonates with sepsis, a clinically critical but understudied population whose unique iron physiology creates a permissive environment for *ybt*-mediated fitness advantages. Neonatal serum is deficient in transferrin (30), weakening nutritional immunity and thus providing a competitive advantage to the *ybt*-producing strains. This neonatal-specific physiology underscores the importance of genomic signatures that can capture pathogen fitness advantages under these conditions. These features underscore why genomic markers capable of capturing pathogen fitness—and not AMR alone—may provide improved discriminatory power. Although murine models are considered gold standards for studying hypervirulent *K. pneumoniae*, they have limitations in replicating human host-pathogen interactions (31). Therefore, genomic definitions of *K. pneumoniae* hypervirulence have been proposed as an alternative to mouse models (32). Our *in vitro* infection model using A549 cells demonstrates *ybt*’s role in human intracellular survival and supports the value of functional assays as an adjunct to genomic association analysis.

Interestingly, Grubwieser et al. (2022) (33) established that *K. pneumoniae* is capable of proliferating intracellularly within airway epithelial cells and that infection can induce ROS production, activating an iron export phenotype in epithelial cells. The authors also suggested that the presence of siderophores might provide an intracellular advantage to *K. pneumoniae*. We observed intracellular proliferation of clinical isolates in A549 cells, suggesting a distinct role in respiratory colonization. Additionally, we observed that *ybt*-positive isolates suppressed ROS generation, which may reduce activation of the epithelial iron-export cassette, enabling the bacteria to retain access to intracellular iron and evade nutritional immunity. This observation complements prior systemic infection models by highlighting the importance of the epithelial niche—an understudied environment in neonatal sepsis pathogenesis.

Siderophore production has previously been identified as an independent predictor of sepsis in *K. pneumoniae* bacteremia (34). Several studies have suggested associations between siderophore-associated virulence factors—especially ybt and/or iuc—and mortality in both neonates (5) and adults (35, 36). Mukherjee et al. (37) documented the presence of *ybt* in isolates from fatal neonatal cases but did not confirm causality. In our cohort, all isolates from fatal cases carried *ybt*, with some also carrying *iuc*. Aerobactin (*iuc*) enhances systemic survival and growth, but unlike *ybt*, it exhibits weaker ferric iron binding (Kf = 10^22.9^ vs 10^36^) (38, 39). Moreover, *ybt* neutralizes oxidative stress by superoxide radicals via Cu²^+^-ybt complexes that mimic superoxide dismutase (40). These biochemical distinctions may partially explain the intracellular fitness advantage of *ybt*-positive strains that we observed.

Isolates from fatal outcomes showed a higher burden of multidrug resistance (MDR/XDR/PDR), particularly to β-lactams and extended-spectrum cephalosporins, and frequently exhibited strong biofilm formation. Genomic profiling revealed convergence of AMR and virulence loci in these isolates, with ST14 (41) and ST231 (42, 43) predominating among non-survivors—lineages previously associated with MDR backgrounds and siderophore acquisition, including *ybt* (37, 44). Antibiotic resistance patterns in our isolates mirror global and Indian trends (5, 16, 45), particularly for β-lactam antibiotics. Amikacin remained more effective than gentamicin (6). Although ESBL and carbapenemase genes, such as *bla*_*CTX*_, *bla*_*NDM*_, and *bla*_*OXA*_ genes, were widespread and mirrored national and global trends (46, 47), these resistance determinants alone did not discriminate outcomes, indicating that resistance is necessary but not sufficient to explain disease severity. Historically, AMR and virulence in *K. pneumoniae* were considered mutually exclusive (8). However, convergent strains now harbor both traits (48, 49). Holt et al. (9) first noted *ybt* in KPC- and ESBL-producing clones, such as ST258 and ST14. Horizontal gene transfer, especially via ICEs and plasmids (50), appears central to this convergence. Recent work implicates ICEkp in *ybt* spread (51). The co-occurrence of *pks* (colibactin), *ybt*A, and MDR has been linked to 30-day mortality in adults (52). Our *ybt*-positive isolates generally harbored ESBL genes and ICEkp lineages. In this cohort, ybt-positive MDR strains were isolated from non-survivors, suggesting that acquisition of *ybt* may contribute to increased clinical severity.

Beyond these individual contributions of AMR, virulence loci, and sequence type, our findings underscore the value of an integrative genomic framework for neonatal sepsis prognostication. Prior genomic approaches, including the recent hvKp definition proposed by reference (32), emphasize predefined virulence plasmid markers but do not provide a data-driven predictive model nor address neonatal infections. In contrast, our exploratory LASSO-derived composite severity model combines siderophore genetics (particularly *ybt*), AMR genotype, phenotypic susceptibility, biofilm behavior, and sequence type into a unified predictive structure. This integrative approach improves discrimination substantially (AUC 0.954) compared with any single genomic or phenotypic marker, suggesting that neonatal pathogenicity arises from the interaction of multiple pathogen-level traits rather than from a single virulence determinant. While preliminary due to sample size, this model illustrates a scalable strategy for neonatal-specific pathogen genomic risk scoring and points to the need for larger, multicenter genomic studies to validate and refine early-warning classifiers for clinical use.

Given the challenges posed by the convergence of antimicrobial resistance and virulence, complementary therapeutic approaches are urgently needed. Siderophores promote bacterial proliferation and modulate host immune responses (29, 33), making them attractive anti-virulence targets. Strategies under development include siderophore-targeting antibiotics, such as cefiderocol (53), anticalins (7), and gallium-based agents (54), although emerging resistance (55) underscores the need for careful evaluation. These approaches are likely to complement rather than replace current empirical regimens recommended by WHO guidelines, which rely on gentamicin with ampicillin or penicillin (56, 57). Addressing this dual threat will require integrated surveillance, infection control, antimicrobial stewardship, and sustained investment in anti-virulence therapeutics.

Together, our genomic, phenotypic, and functional analyses highlight *ybt*-associated siderophore biology, sequence type, and other pathogen-level traits as potential determinants of neonatal outcome. The exploratory LASSO-derived model illustrates a scalable framework for integrating genomic signatures into neonatal sepsis risk stratification. Larger, multi-center genomic data sets will be essential for validating and refining such pathogen-based prognostic tools. These findings demonstrate the value of combining genomic and functional approaches to identify clinically relevant pathogen determinants in neonatal *K. pneumoniae* sepsis.

### Study limitations

Despite comprehensive genomic and functional analyses, this study is limited by a small sample size, which constrains statistical power and restricts the generalizability of the predictive modeling. The absence of detailed host-level clinical metadata limits the ability to disentangle pathogen effects from host susceptibility. The A549-based *in vitro* assays, while informative for epithelial interaction, cannot fully recapitulate the complexity of host-pathogen dynamics in neonatal sepsis and underscore the need for deeper mechanistic investigation using more physiologically relevant models. External validation with larger, multi-center neonatal cohorts will be essential to refine and validate the exploratory genomic severity model proposed here.

### Conclusion

Neonatal *K. pneumoniae* sepsis in this cohort was predominantly caused by MDR strains, with isolates from non-survivors showing enhanced biofilm formation, ybt carriage, and superior adhesion and intracellular proliferation in epithelial cells. Integrating genomic signatures with functional phenotypes, our exploratory predictive model identified ybt and specific sequence types as pathogen-level features associated with clinical outcome. Together, these findings support a contributory role for ybt-associated biology in virulence and demonstrate the value of combined genomic and functional approaches in identifying potential determinants of neonatal sepsis severity. Larger cohorts will be required to validate and extend this integrative framework toward clinically actionable risk stratification.

## Data Availability

The data have been deposited with links to BioProject accession number PRJNA1250426 in the NCBI BioProject database (https://www.ncbi.nlm.nih.gov/bioproject/).
